# A Growing Vice

**Published:** 1875-10

**Authors:** 


### A GROWING VICE.

"I am a strong man," said a physician, "and have repeated opportunities to test my resolution under the most trying circumstances, yet so insidious and treacherous is the habit of using opium, once contracted, so demoralizing in its influence on the power of the will, that I would not dare to trust myself to a continued use of the drug even for a limited time. The craving it creates directly destroys all power of resistance. I have known ladies who in their advanced age had contracted the habit, and who carried the poison about their persons in the form of salts of opium, using it almost hourly. Indeed, the vice is more prevalent among women than among men."

With regard to the superinducing causes of the evil a distinguished physician says: They may be divided into two classes, the physical and the moral. Under the first head should be placed all persons suffering from neuralgia, rheumatism, dysenteric affections, tic doloureux, consumption, hysteria, uterine affections, &c. In such cases opium in some form is usually first prescribed by physicians, and continued by the patient after the habit has been acquired. The moral causes are often pecuniary embarrassments, disagreements, jealousies, sensualism, &c. In these cases the drug is resorted to to drown thought or promote excitement.

" There is no doubt that opium is too miscellaneously prescribed, perhaps to the extent that if it did not exist the human race would be better off. It is a most useful therapeutic agent, but of all the articles of the *materia medica* it requires the most intelligence and care in its use. Patients suffering with delirium tremens have been killed by it; and yet in the same disease, with proper caution, it is a most beneficial remedial agent. In gunshot wounds, in railroad accidents, and in excessive hemorrhages, opium, if properly administered, is a boon to mankind; but if thrown into the system injudiciously, it is most disastrous to human life.

Then the physical prostration produced by the use of opium renders its victims liable to contagion, unless they are continually under the influence of the drug. The sudden discontinuance of the use of the drug has a somewhat similar effect to that produced by a discontinuance of the excessive use of alcoholic stimulants in the case of confirmed inebriates. It is followed by a sort of mental collapse, or great prostration of the mental energies. Opium gives and takes away. It defeats the steady habit of exertion, and creates spasms of irregular effort. It ruins the natural power of life; it develops preternatural paroxysms of intermitting power; it brings on hypertrophy or enlargement of the liver, habitual constipation, bronzed complexion, rigidity of skin, vacancy of expression, general hyperæsthesia, and insomnia, or sleeplessness; a morbid condition of the stomach, rejecting many kinds of food that are regarded by medical men as simple and easy of digestion; acute, shooting pains, that are confined to no one part of the body; and unnatural sensitiveness to cold, frequent cold perspirations in parts of the body, and a chronic tendency to impatience and irritability of temper, with paroxysms of excitement wholly foreign to the natural disposition. In the long run it saps the vitality. It engenders sterility. The offspring, if there be any, is generally marked by organic cerebral defects—impaired intellect, and constitutional cachexia, or deteriorated vitality.

The amount of opium imported into the United States in 1874 was about two hundred tons; and it is estimated that five per cent of the opium sold by retailers would cover all the prescriptions of physicians proper, and five per cent more excepted from the entire as an extra allowance for the various nostrums afloat, would be liberal and abundant. This is from a comparison of opinions entertained by many apothecaries of New York city, and to these estimates the experienced Dr. Carnochan adds the opinion that while the therapeutic value of opium has suffered no abatement in the estimation of the profession, the total of prescriptions is proportionately less than it was twenty years ago.

